# Supplementary material for: Identification of a pathogen causing fruiting body rot of Sanghuangporus vaninii
Source: PeerJ. 2023 Sep 4;11:e15983. doi: 10.7717/peerj.15983 (PMC10484203; doi:10.7717/peerj.15983)
Supplement: Supplemental Information 1 — The species, strains and GenBank accession numbers of 31 Trichoderma and 1 Sphaerostilbella lutea. The ITS sequences of these strains were used in the phylogenetic analysis. [file peerj-11-15983-s001.docx]

| Species | Strains | GenBank Accession No. |
| --- | --- | --- |
| *Trichoderma lentinulae* | CGMCC 3.19847 | MN594469 |
| *Trichoderma rifaii* | CBS 130746 | NR_137305 |
| *Trichoderma neotropicale* | CBS 130633 | MH865818 |
| *Trichoderma peberdyi* | CEN 1426 | MK714906 |
| *Trichoderma linzhiense* | HMAS 248846 | NR_154575 |
| *Trichoderma tomentosum* | DAOM 178713a | NR_134357 |
| *Trichoderma atrobrunneum* | CBS 548.92 | NR_137298 |
| *Trichoderma lixii* | CBS 110080 | NR_131264 |
| *Trichoderma simile* | YMF 1.06201 | MN977793 |
| *Trichoderma xiciacum* | HMAS 248253 | NR_171952 |
| *Trichoderma pinicola* | KACC 48486 | NR_173781 |
| *Trichoderma harzianum* | CBS 226.95 | AY605713 |
| *Trichoderma inhamatum* | CBS 273.78 | MH861134 |
| *Trichoderma simmmonsii* | CBS 130431 | NR_137297 |
| *Trichoderma zeloharzianum* | YMF 100268 | NR_165872 |
| *Trichoderma aggressivum* | DAOM 222156 | AF443911 |
| *Trichodermia pleuroticola* | CBS 124383 | NR_134420 |
| *Trichoderma ammazonicum* | CBS 126898 | MH864268 |
| *Trichoderma pleuroti* | CBS 124387 | MH863369 |
| *Trichoderma solum* | HMAS 248848 | NR_154576 |
| *Trichoderma tawa* | BPI 745837 | NR_138430 |
| *Trichoderma virens* | CBS 249.59 | MH857855 |
| *Trichoderma crassum* | DAOM 164916 | NR_134370 |
| *Trichoderma longipile* | CBS 340.93 | MH862410 |
| *Trichoderma tropicosinense* | HMAS 252546 | NR_134441 |
| *Trichoderma spirale* | DAOM 183974 | NR_077177 |
| *Trichoderma floccosum* | CBS 124372 | NR_137306 |
| *Trichoderma barbatum* | CBS 125733 | MH863657 |
| *Trichoderma viride* | CBS 119325 | NR_138441 |
| *Trichoderma lanuginosum* | CBS 125718 | MH863649 |
| *Trichoderma medusa* | CBS 125719 | MH863650 |
| *Sphaerostilbella lutea* | CBS 405.59 | MH857905 |
